# Zero-current chronopotentiometry for wired biosensors

**DOI:** 10.1007/s00604-025-07769-8

**Published:** 2025-12-16

**Authors:** Andrea Nonis, Polyxeni Damala, Eric Bakker

**Affiliations:** Department of Mineral and Analytical Chemistry, University of Geneva, Quai-Ernest 30, Geneva, 1205 Switzerland

**Keywords:** Zero-current chronopotentiometry, Glucose determination, Wired biosensor, Redox polymer

## Abstract

Enzyme biosensors used in continuous monitoring applications rely on amperometry as redout, which continuously draws energy, introduces ohmic drops and requires a reasonably reliable reference electrode. To help address these challenges a new experimental “zero-current” chronopotentiometry readout for wired glucose biosensors is introduced. The method records the transition time needed to chemically reduce the wired redox probe at the electrode by the enzymatically catalyzed turnover of glucose. After an electrochemical oxidation step, the open circuit potential changes over time because the enzymatic turnover continuously reduces the redox probe, thereby altering the ratio of reduced to oxidized form at the electrode. The exhaustion of the oxidized probe is indicated by an inflection of the potential at a specific transition time. The inverse value of the transition time is found to be proportional to glucose concentration, consistent with mass transport-limited kinetics across the diffusion limiting membrane and serves as sensor signal. For this reason, the specific potential value is not important for the measurement, allowing one to employ less accurate reference electrodes. Lower glucose concentrations result in longer transition times, resulting in a method that successfully covers the entire physiological concentration range.

**Graphical Abstract:**

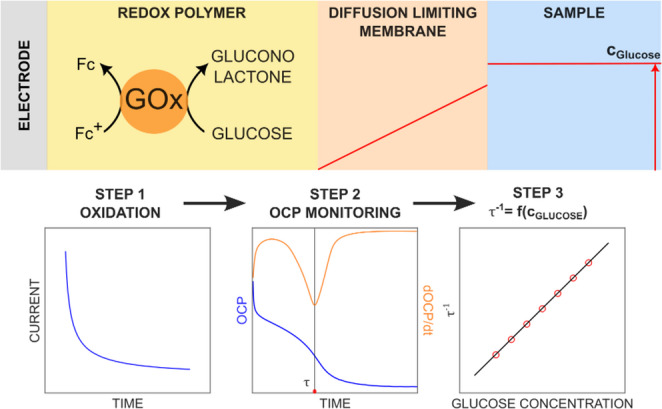

**Supplementary Information:**

The online version contains supplementary material available at 10.1007/s00604-025-07769-8.

## Introduction

Glucose testing is ubiquitous around the world and performed either as single measurements on discrete samples or, more recently, by continuous glucose monitors (CGM). Most of such tests rely on enzyme-based amperometric measurements in which a faradaic current is observed as signal. This current originates from electron transfer between the enzyme and a redox mediator at the electrode [1, 2]. Introduced by Heller and co-workers, wired-enzyme biosensors achieve an electrical connection between the electrode surface and the enzyme via redox moieties grafted on a polymer that entraps the enzyme [3, 4]. This direct electron shuttling is used in many sensors and fuel cells because of its high current density [5–7]. Amperometry is attractive for single-use point of care measurements and provide robust current signals that are directly proportional to analyte concentration. Nevertheless, limitations for CGM arise from multiple sources; biofouling from foreign body response [8, 9], enzyme loss of activity by local acidification from byproducts (H_2_O_2_ and gluconic acid) [10, 11] and the continuous charging current of redox moieties not directly connected to the enzyme [12]. The first two can be addressed with different enzyme encapsulation or sensing strategies [11, 13], while the third one gives an undesired constant current drift. For this reason they are operated continuously, rather than intermittently, to minimize the influence of this parasitic current [2]. Additional drawbacks are the occurrence of an ohmic drop of the measurement cell that may change in unpredictable ways and that impacts the value of the effectively applied potential at the working electrode. Similarly, the reference electrode must be sufficiently reliable to avoid the application of a potential outside the limiting current range.

Passive electrochemical techniques that operate at zero current would overcome some of these limitations. A zero-current method was first proposed by Nagy and co-workers in which the analyte NADH continuously reduces the electrode mediator, resulting in a change in the slope of the open circuit potential (OCP) with time as a function of the analyte concentration [14]. Yarnitzky and co-workers described the same concept using a wired enzyme-based glucose biosensor in which the electrons generated by the enzymatic oxidation will reduce the redox polymer, here recording the OCP as signal at a predefined time [15]. More recently, Muhs and co-workers used the same principle in a quasi-equilibrium condition for an “off-on” glucose biosensor for CGM [13, 16].

In contrast to the above contributions, the approach proposed here does not depend on the actual value of the potential or slope but only identifies the time at which the potential is changing in a characteristic manner. This new zero-current chronopotentiometric method for wired biosensors uses the transition time ($\:\tau\:$) as signal. This approach is free of undesired charging currents and largely independent of the quality of the reference and counter electrodes since neither a current nor potential amplitude is relevant for the signal, only its characteristic change with time. To limit the enzyme conversion rate by the glucose mass transport rather than the enzyme itself, the enzyme layer is overcoated with a diffusion limiting membrane [12]. An anodic potential is first applied to pre-oxidize the redox moieties of a cross-linked polymer containing the glucose oxidase enzyme (GOx) (step 1). The open circuit potential is then monitored with time at zero-current until the potential is found to inflect (step 2), giving the transition time. The inverse of the transition time is used as signal and is correlated to the glucose concentration (step 3).

## Methods

### Chemicals

Glucose Oxidase from Aspergillus niger (type VII, lyophilized powder, ≥ 1000000 U/g solid) (GOx), (6-bromohexyl) ferrocene, poly(ethylene glycol) diglycidyl ether (average Mn ~ 500) (PEGDE), branched polyethylenimine (Mw ~ 25000, Mn ~ 10000) (bPEI), methanol (reagent grade 99%) (MeOH), acetonitrile (reagent grade 99% ) (MeCN), Nafion perfluorinated resin solution (5% wt. in lower aliphatic alcohols and water, 15–20% water), D-(+)-glucose (≥ 95%), potassium chloride (KCl), sodium phosphate dibasic (ACS reagent, ≥ 95%) (Na_2_HPO_3_) and sodium chloride (puriss, ≥ 95%) (NaCl) were purchased from Sigma Aldrich. Potassium phosphate monobasic (99 + %, extra pure) (KH_2_PO_3_) was purchased from Acros Organics. Hydrochloric acid (1 M, NIST Standard Solution) was purchased from Fisher Chemical.

Diluted 0.5% wt. Nafion solution was made by dilution of the 5% wt. solution in Milli-Q water. The diluted 1:10 cross-linker solution was made by diluting PEGDE in Milli-Q water. All other aqueous solutions were prepared in Milli-Q water (18.20 MΩ cm). Phosphate buffered saline solution (PBS) (pH 7.49) was made with a concentration of 137 mM sodium chloride, 2.7 mM potassium chloride, 10 mM sodium phosphate dibasic, 1.8 mM potassium phosphate monobasic prepared from the respective salts. The glucose standard used for all experiments were made by dilution in PBS of a previously made 1 M glucose in PBS solution.

### Instrument and measurements

All electrochemical measurements were performed with an Autolab PGSTAT128N and PGSTAT204 controlled with Nova 2.1.4 software (Metrohm Switzerland). A three-electrode configuration was used, consisting of a platinum wire as counter electrode (CE), an Ag/AgCl wire as reference electrode (RE) and an in-house build glassy carbon electrode as working electrode (WE) (Fig. S1b, c). The glassy carbon (GC) working electrode was fabricated by encasing a GC rod ($\:\varnothing\:$ 2 mm) in a polyether ether ketone (PEEK) tube. All the measurements were made inside an in-house build flow cell (Fig. S1) with an IC pump (high precision multichannel dispenser, ISTEMATEC) at constant flow (255 or 510 µL min^− 1^) excepted the zero-current chronopotentiometric measurements when no diffusion limiting membrane is present.

Cyclic voltammetry was performed between − 0.100 and 0.600 V at a 0.050 V s^− 1^ scan rate in all cases except for the scan rate study where the scan rate used were 0.010, 0.025, 0.050, 0.075, 0.100, 0.250, 0.400 and 0.600 V s^− 1^. For amperometry measurements, a fixed potential of 0.400 V was applied. For “zero-current” chronopotentiometric measurements, the redox polymer was first completely oxidized by applying a constant 0.400 V potential for 45 s. Then the open circuit potential (OCP) was measured as a function of time until a cut-off potential was reached. The time corresponding to the potential drop due to the conversion of all oxidized ferrocene is determined using the lowest point of the corresponding derivative of potential trace in function of time and used as signal. Five chronopotentiometric replicates were performed for each solution and only the last three were used to obtain the mean and standard deviation. When changing solution, the flow cell was flushed with at least three times the cell volume of the new solution before doing the next measurement. For the O_2_ influence experiment, the measurements were first made with normal glucose solutions, then the oxygen was removed by bubbling N_2_ for 30 min in the same solutions.

The thickness of the Nafion layer was measured with a Olympus BX40 optical microscope equipped with a 3CCD color vision camera module using a 50x magnification lens. The picture was analyzed with ImageJ. The Nafion membrane was deposited on a cover glass slide covered with tape in the same way as on the biosensor (see Biosensor Preparation). Once the membrane dried, it was split in half with a razor blade to obtain a cross section of the membrane on the taped cover glass slide. The cross section was then measured.

### Synthesis of the Fc-C_6_-bPEI polymer

The Fc-C_6_-bPEI used was synthetized accordingly to a previously published synthesis by our group with a substitution degree of 10.7% and a ferrocene concentration in the polymer of 1.49 mmol mg^− 1^ (1.49 M) [12].

^1^H-NMR (400 MHz, CDCl_3_): 4.15–3.95 (m, Cp-Fe-Cp-**H**), 3.80–2.20 (m, broad, -C**H**_2_-C**H**_2_-NH-, Fc- C**H**_2_-(CH_2_)_5_-, Fc-(CH_2_)_5_-C**H**_2_-), 1.55–0.95 (m, Fc-(CH_2_)n-C**H**_2_-(CH_2_)m), 0.92 − 0.75 (m, Fc-(CH_2_)n-C**H**2- (CH_2_)m).

### Biosensor preparation

A 20 mg mL^− 1^ redox polymer solution (pH ~ 5) was prepared by dissolving 8 mg of Fc-C_6_-bPEI in 400 mL of Milli-Q water and 0.1 M HCl solution. A Fc-C_6_-bPEI/GOx/cross-linker solution was prepared by mixing 28 µL of the polymer solution, 12 µL of a 50 mg mL^− 1^ GOx solution and 1.5 µL of 1:10 diluted cross-linker solution. Once mixed, 2 µL of this cocktail were drop casted on the GC working electrode (previously polished with 0.3 μm alumina powder) and left to cure overnight at room temperature. Three layers (2 µL each) of 0.5% wt. Nafion solution were drop cast on the modified electrode, with a 30 min drying period between each addition. After the last layer, the biosensor was left to dry at room temperature for 3 h before using.

### Zero-current chronopotentiometric simulation

The numerical zero-current chronopotentiometric simulations were made using Wolfram Mathematica 13.2. The system was considered as one dimensional diffusion problem using a finite element treatment (see Supporting Information) [17, 18]. Briefly, a fixed depletion rate was imposed as boundary condition at the redox polymer interface. This imposed rate is calculated from the glucose fluxes obtained from the zero-current chronopotentiometry calibration (Table S2). The second boundary condition at the electrode surface was treated as an inert boundary. In the bulk of the polymer, the concentration was let to evolve using diffusional mass transport in one dimension. The duration of the simulation corresponds to the measured transition time.

## Results and discussion

### Theoretical model

The enzyme converts glucose to gluconolactone by being itself reduced:

1 $$\:Glucose+GOxFAD\:\to\:Gluconolactone+GOxFAD{H}_{2}$$

It will subsequently be re-oxidized by the ferrocenium ions ($\:{Fc}^{+}$) of the polymer:

2 $$\:GOxFAD{H}_{2}+2{Fc}^{+}\:\to\:GOxFAD+2Fc+2{H}^{+}$$

While the enzyme is continuously recycled in this manner, the oxidized ferrocene will gradually become exhausted in the absence of current flow. The redox polymer at the electrode dictates the electrode potential, which will change with time as a consequence of enzyme turnover. The ferrocene/ferrocenium mole ratio imposes the potential via the Nernst equation:

3 $$\:E={E}_{F{c}^{+}/Fc}^{0}-\frac{RT}{F}ln\left(\frac{{n}_{Fc}}{{n}_{{Fc}^{+}}}\right)$$

where $\:{E}_{F{c}^{+}/Fc}^{0}$ is the standard potential of the ferrocenium/ferrocene redox couple, $\:{n}_{Fc}$ the number of moles of ferrocene moles and $\:{n}_{{Fc}^{+}}$ the number of moles of ferrocenium. At the transition time $\:\tau\:$, all ferrocenium is consumed and a potential drop is observed. Beyond $\:\tau\:$, another redox couple will dictate the potential and Eq. 3 should be modified accordingly. When a diffusion limiting membrane is present, $\:\tau\:$ is related to glucose concentration $\:{c}_{Glu}$ and used as signal (see Supporting Information for details):

4 $$\:\frac{1}{\tau\:}=\frac{2{K}_{m}A}{{n}_{Fc}^{T}{\delta\:}_{m}}{c}_{Glu}$$

where $\:{K}_{m}={P}_{m}{D}_{m}$ is a constant, with $\:{P}_{m}$ the glucose partition constant from aqueous to membrane phase and $\:{D}_{m}$ the glucose diffusion coefficient in the diffusion limiting membrane. $\:A$ is the electrode area, $\:{n}_{Fc}^{T}$ is the electrochemical accessible total number of moles of ferrocene, and $\:{\delta\:}_{m}$ is the membrane thickness. Ideally, the signal ($\:{\tau\:}^{-1}$) is a direct function of the ferrocene conversion rate, which is equal to the glucose flux if membrane diffusion, rather than enzyme kinetics, is the rate limiting step. In analogy, a different relationship between $\:\tau\:$ and $\:{c}_{Glu}$ is expected in absence of diffusion limiting membrane in which the diffusion thickness increases approximately with $\:{\delta\:}_{aq}=2\sqrt{{D}_{aq}t}$ (see Supporting Information for details):

5 $$\:\frac{1}{\sqrt{\tau\:}}=\frac{A\sqrt{{D}_{aq}}}{{n}_{Fc}^{T}}{c}_{Glu}$$

Where $\:{D}_{aq}$ is the diffusion coefficient of glucose in water.

### Model confirmation

**Fig. 1:**
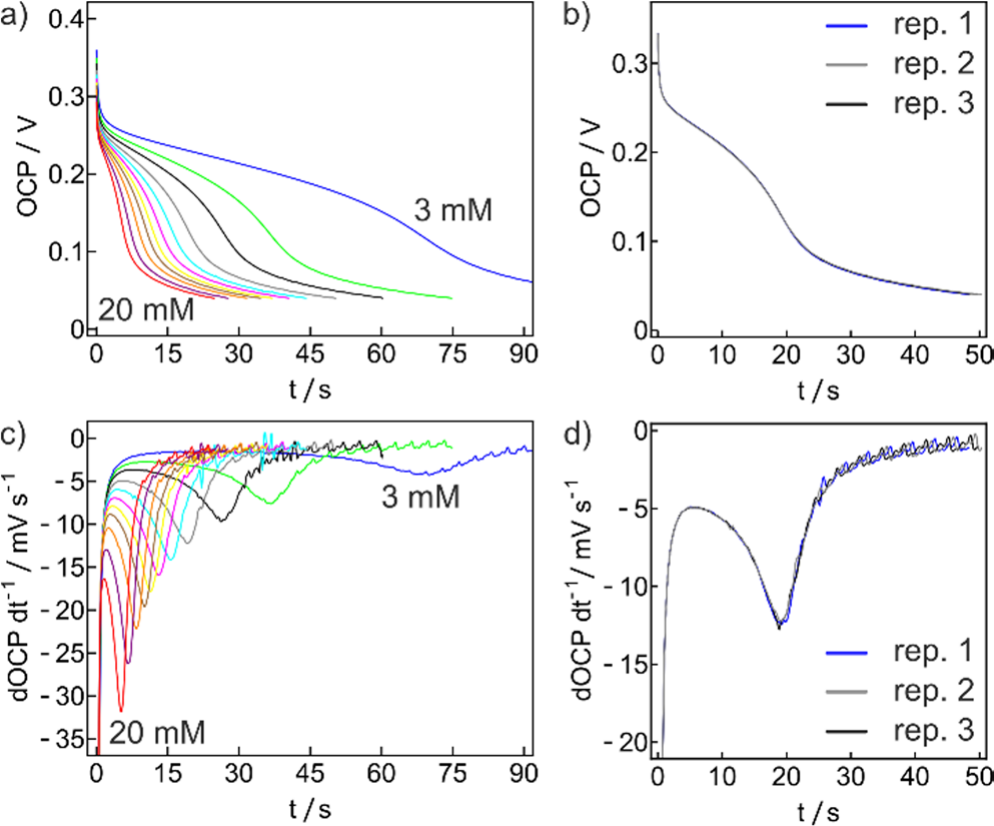
(**a**) Zero-current chronopotentiometric time traces for 3 to 20 mM glucose. (**b**) Reproducibility of zero-current chronopotentiometric time traces for 6 mM glucose. (**c**) Time derivatives of the data shown in a. (**d**) Time derivatives of the data shown in b

**Fig. 2:**
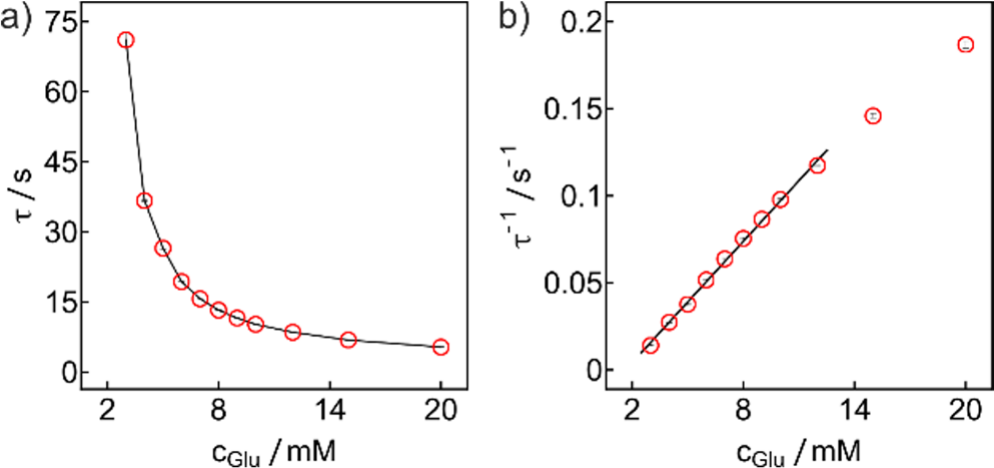
(**a**) Experimental glucose calibration shown as transition times τ as a function of glucose concentration. (**b**) Corresponding calibration curve expressed as the inverse of the transition time τ as a function of glucose concentration

The concept was demonstrated with a cross-linked ferrocene-modified branched polyethylenimine entrapping the enzyme GOx (Fc-C_6_-bPEI/GOx/PEGDE) sensing layer on a glassy carbon electrode. The Fc-C_6_-bPEI/GOx/PEGDE modified electrode overcoated by a three-layer Nafion diffusion-limiting membrane was placed in a flow cell made in house with constant flow (Figs. S1, S2). An anodic potential of 0.400 V was applied for 45 s to oxidize the redox polymer (Fig. S3) followed by monitoring the OCP with time until a potential cutoff value was reached. With the optimized biosensor, a zero-current chronopotentiometric glucose calibration was obtained in the range of 3–20 mM glucose. For each concentration a series of five repetitions were performed. The first two points were found to give somewhat longer transition times, which was attributed to the time required to reach steady-state across the polymer. For this reason the average and standard deviation of the transition time were calculated from only the last three repetitions (Fig. 1a) as they exhibited appropriate steady state conditions. The transition time $\:\tau\:$ was determined from the first derivative of OCP over time (Fig. 1c) and its reciprocal was plotted as a function of glucose concentration (Fig. 2b).

A linear relationship to the reciprocal of the transition time with a slope of 0.0116 ± 0.0002 s^− 1^ mM^− 1^ was observed in the 3–12 mM range. Beyond 12 mM a deviation from linearity was observed that may indicate the onset of limiting enzyme kinetics.

Considering a total thickness of $\:{\delta\:}_{m}$ = 45.5 μm, the thickness of the sensing layer (22.5 ± 3.0 μm) and that of the Nafion membrane (23.0 ± 0.9 μm) (see Fig. S4), a value of $\:{K}_{M}$ = 1.12 × 10^−7^ cm^2^ s^−1^ is estimated, which is reasonably close to that obtained from Randels-Sevcik and Cottrell experiments, 8.75 ± 0.27 × 10^−7^ cm^2^ s^−1^ and 2.68 ± 0.47 × 10^−7^ cm^2^ s^−1^ respectively (Figs. S6, S7). It shows comparable diffusion order between the electrons and glucose inside the sensor, suggesting an influence of the transport of electrons and their corresponding counter ions on the transition time.

**Fig. 3:**
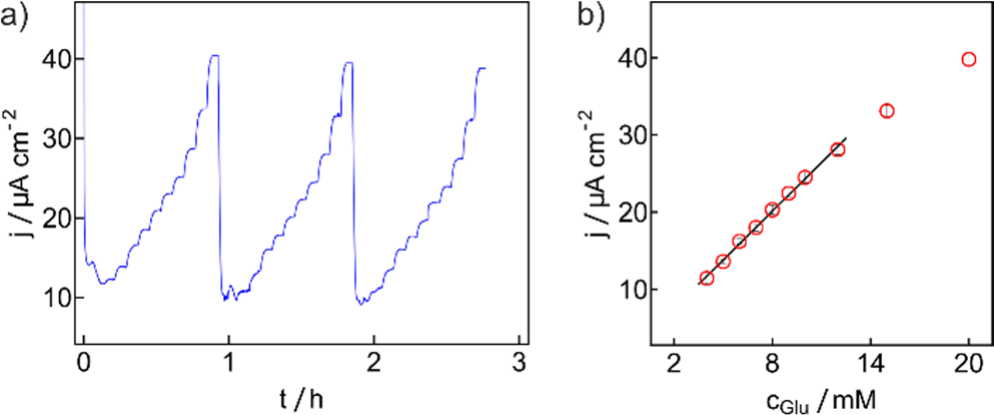
Chronoamperometric glucose calibration using the same biosensor used for zero-current chronopotentiometric calibration in Fig. 1. (**a**) Raw chronoamperometric traces of current density as a function of time. (**b**) Averaged current densities for the three replicates in a) as a function of glucose concentration (error bars are standard deviations)

Chronoamperometric calibration was linear between 4 and 12 mM glucose (slope = 1.86 ± 0.07 µA cm^− 2^ mM^− 1^). Perhaps surprisingly, the zero-current chronopotentiometry exhibits a higher reproducibility (Fig. 1b, d) and lower standard deviation compared to the corresponding amperometric calibrations using the very same sensor (Fig. 3): calculated precision for zero-current chronopotentiometry is 0.05 mM, 6 times lower than for chronoamperometry (0.29 mM). This may be attributed to the passive readout of the new method, unaffected by parasitic background currents. From Table S1, the charges for the oxidation step for each replicate gave standard deviations of < 1%, supporting the reproducibility of the zero-current chronopotentiometric measurements (Fig. 1b, d). As expected, similar glucose fluxes for the oxidation step and the amperometric calibration were observed as shown in Fig. S8.

**Fig. 4:**
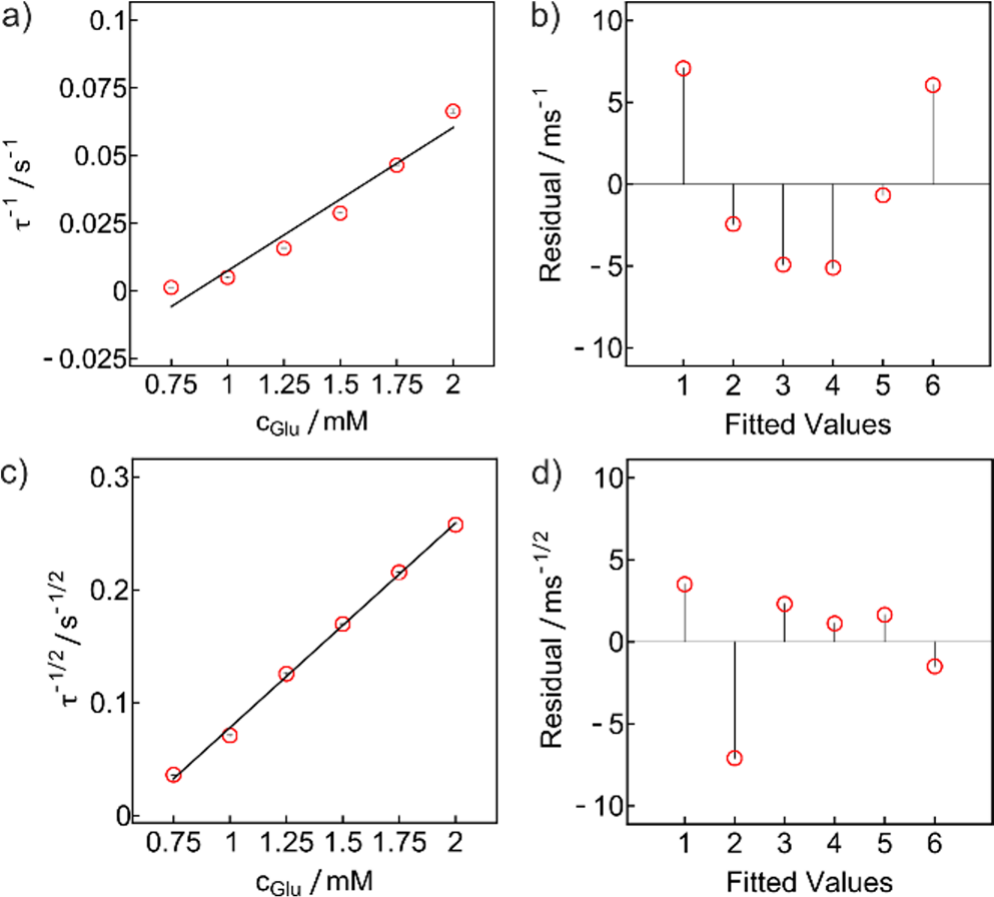
(**a**) Zero-current chronopotentiometric calibration from 0.75 to 2 mM glucose without Nafion membrane using the reciprocal of τ and (**b**) the corresponding residuals, and (**c**) the reciprocal of the square root of τ with (**d**) the corresponding residuals

The applicability of Eq. 5 is also demonstrated with the same zero-current chronopotentiometric measurements without the diffusion limiting membrane and no solution flow to obtain a quiescent solution (Fig. 4). A distinct linear relationship with the reciprocal of $\:\sqrt{\tau\:}$ was confirmed from the residuals plot, in qualitative agreement with Eq. 5, with a slope of 0.181 ± 0.004 s^− 1/2^ mM^− 1^ (Fig. 4c and d). A residuals plot for τ^−1^ gave a parabolic shape compared to the one for τ^−1/2^ that exhibits random deviations (Fig. 4a and b). Since no diffusion membrane is present, a lower glucose concentration range was needed to be in the range of Michaelis-Menten linearity (Fig. S10) without being limited by the enzyme rate, making Eq. 5 valid.

**Fig. 5:**
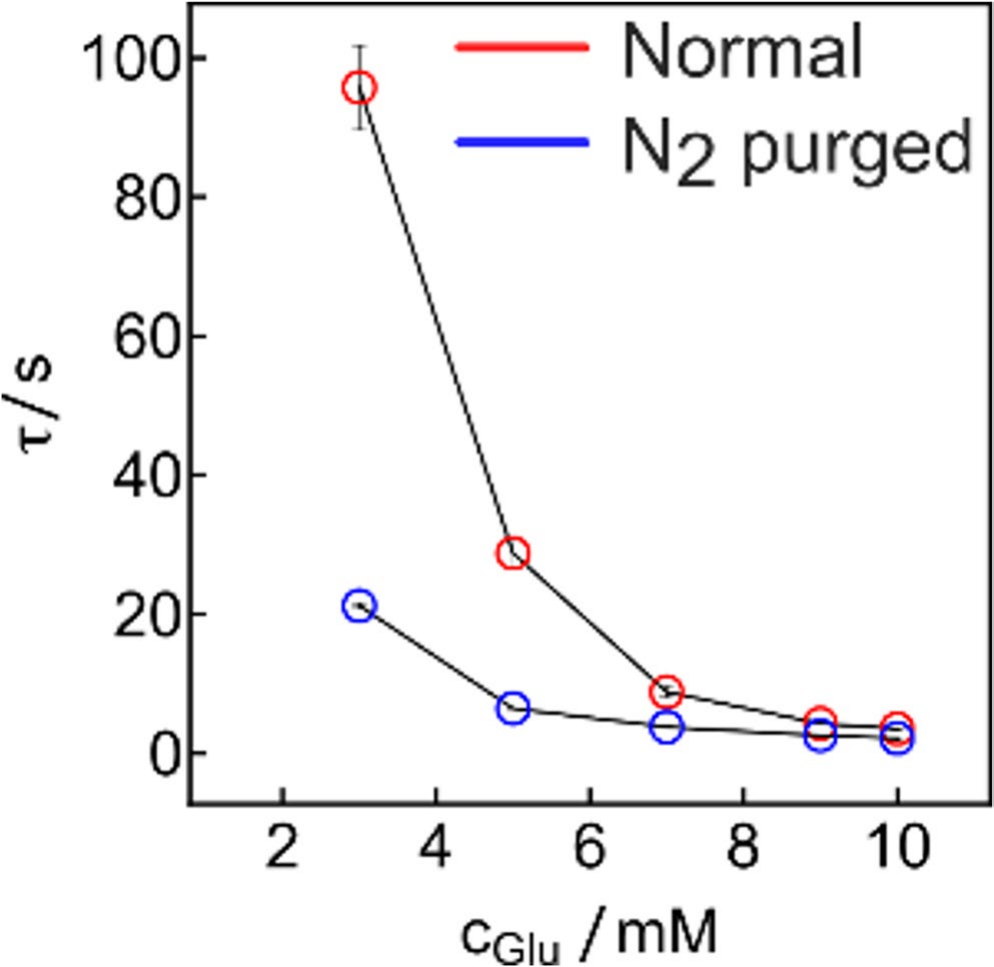
Transition times observed for the atmospheric glucose solution (red, upper curve) and the solution purged for 30 min by N_2_ (blue, lower curve)

The effect of dissolved O_2_ may be important since it will be present in a physiological sample and regenerate the glucose-reduced GOx, competing in this manner with the Fc^+^ moieties. As can be seen, Fig. 5 shows shorter $\:\tau\:$ upon purging of O_2_ with N_2_. This indicates some competition between the two electron acceptors, resulting in longer transition times in the presence of dissolved O_2_ [19, 20]. This effect is most pronounced at lower glucose concentration (below 7 mM). Above this level, the difference between purged and unpurged solution becomes less important since a given amount of dissolved O_2_ will have an ever-smaller parasitic effect on electron turnover. In physiological samples, oxygen fluctuations may affect the sensor response as expected for glucose oxidase-based sensors. This may be greatly mitigated by using a glucose dehydrogenase enzyme and optimizing the polymer-enzyme ratio [20].

**Fig. 6:**
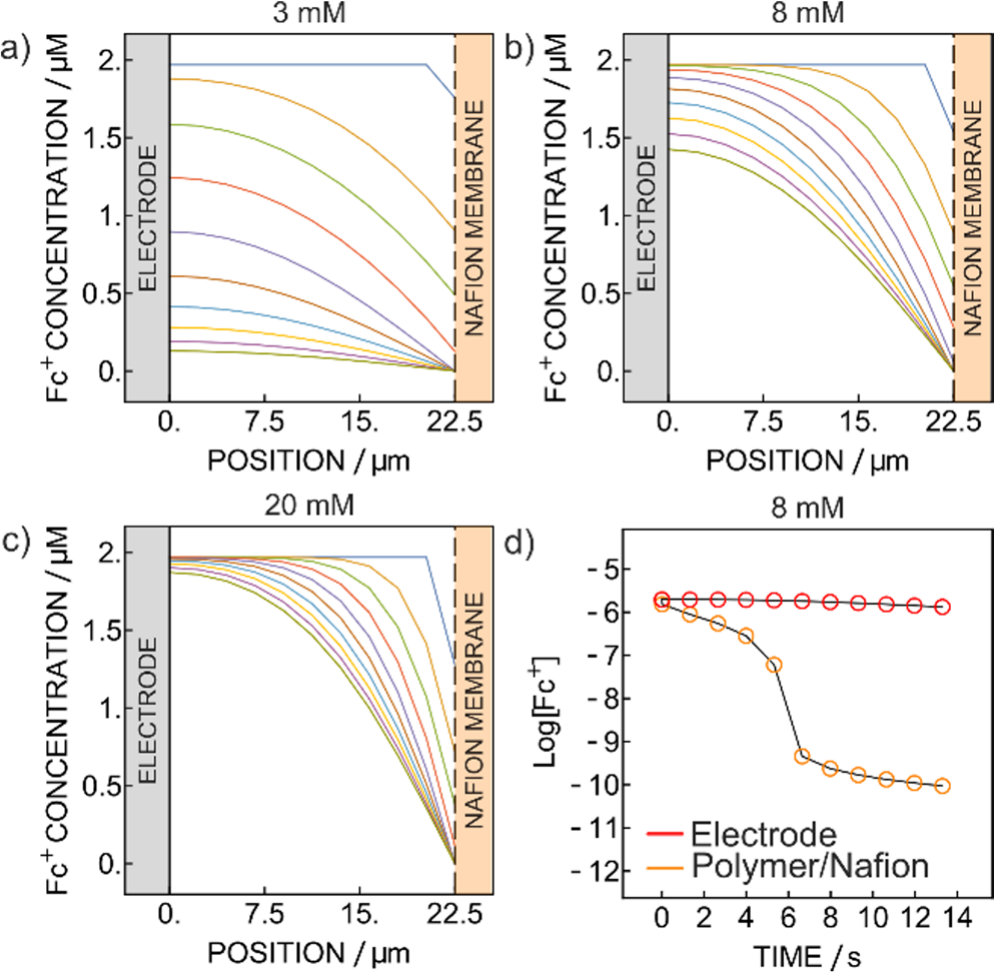
Simulated zero-current chronopotentiometric Fc^+^ concentration profile inside the sensing layer for (**a**) 3 mM (**b**) 8 mM and (**c**) 20 mM (**d**) Logarithm of the Fc^+^ concentration as a function of time at the polymer/Nafion membrane interface (orange, lower curve) and at the electrode surface (red, upper curve). The simulation was based on the obtained membrane constant $\:{K}_{m}$ from the calibration in Fig. 2

Fluctuations in oxygen levels will not alter the applicability of Eqs. 4 and 5, only the observed slope. As with amperometric biosensors, other interferences from redox active species present in physiological samples, including ascorbic acid or uric acid, may also reduce the ferrocenium ion. This would result in shorted measured times that could be interpreted as excessive glucose concentrations. To avoid this, different encapsulations strategies may be used [21–23]. In Fig. 5 the transition time at 3 mM (95.7 s) is found to be higher than the one shown in Fig. 1 (71.1 s). The difference between those two values from two different sensors is explained by experimental variations from manual film casting.

From Eq. 4, using the $\:{K}_{m}$ value obtained from the slope, the predicted transition times are found to be somewhat shorter than the experimental ones (Table S3).

Chronopotentiometric simulations of the oxidized ferrocene concentration profile inside the sensing layer (Figs. 6, S11, S12) were made using the experimental concentration of ferrocene, $\:{K}_{m}$, τ, and sensing layer thickness. In the time interval of the experimental transition time, the concentration does not reach exactly zero at the electrode surface (Figs. 6, S11, S12). For 3 mM (Fig. 6a) the Fc^+^ concentration almost reached 0 at the electrode but at higher concentration of glucose, it will vary less (Figs. 6d, S12). From the logarithm of the concentration at the electrode surface and at the polymer/Nafion interface are plotted against time (Fig. 6d), we may see that there is a time delay in the concentration variation between the one at the electrode surface and the polymer/Nafion interface owing to the diffusion distance separating the two. This delay is present even with increasing glucose concentration (Fig. S12). Additionally, apparent diffusion coefficients for glucose and that for charge transport (1.12 × 10^− 7^ cm^2^ s^− 1^ and 8.75 × 10^− 7^ cm^2^ s^− 1^, respectively) are similar. This suggests that charge transport kinetics may need to be included in the model to obtain precise estimations of transition times. In the current model, charge transport is not formally considered and instead forms part of the apparent $\:{K}_{m}$ value, which may be an oversimplification.

Additionally, the model considers complete oxidation of glucose as soon as the glucose reaches the sensing layer. Assuming no glucose penetration into the layer may be a general oversimplification. Indeed, the simulation of Fig. 6 suggests that the period for the measured transition time is not sufficiently long for total depletion of oxidized ferrocene at the electrode surface to occur if one assumes that the electrons are only released at the polymer/Nafion interface. The faster transition times observed experimentally indicate glucose turnover deeper inside the sensing layer. From Fig. 6a (3 mM glucose), the concentration at the electrode surface almost reaches 0 while in Fig. 6c (20 mM glucose), the expected decrease is too small to be detectable in the observed transition time. This suggests an effective variability of the membrane thickness $\:{\delta\:}_{m}$ not considered in Eq. 4. While the complications of mass and charge transport kinetics must not be overlooked, it will not affect the basic relationship between transition time and glucose concentration put forward here. The thickness of the sensing layer becomes important as it will affect the transition time. Considering a sensing layer with the same enzyme concentration but changing thickness, a thicker sensing layer should result in a longer transition time while thinner layers should shorten it. With a miniaturized an implanted sensor, this would lead to faster transition times, thereby increasing the temporal resolution of the sensor.

## Conclusions

While continuous glucose sensors tend to be universally operated by constant potential chronoamperometry, this contribution shows that zero-current chronopotentiometry may be a plausible alternative. Quantification is performed with transition time, rather than a current amplitude. The method does not require a prolonged signal stabilization time as in amperometry. Also, since the technique is based on a potential change and not an exact value, the quality of the RE is not as important as in amperometry as long as it is stable for the duration of the measurement. This allows for faster measurement times and intermittent, power saving sensor operation. Compared to amperometry, limiting the perturbation to a short oxidation pulse will likely increase the life span of the sensor by limiting the degradation of the sensing layer. With this zero-current readout some inherent drawbacks, including charging currents, ohmic drops and current drift are avoided, resulting possibly in improved precision and lower power consumption relative to constant potential chronoamperometry. It is worth mentioning that it is critical for this method to use a sufficiently stable redox mediator since the signal is directly linked to its quantitative conversion. The method relies on intermittent measurements, and even if they are closely spaced in time, it cannot be considered a continuous method. An advantage for continuous monitoring devices is the lack of a prolonged conditioning or warm-up time while the sampling frequency on the order of one data point per minute remains diagnostically relevant. This readout principle may be extended to most other types of redox enzymes if they are wired to an appropriate redox polymer that can be oxidized/reduced by the enzymatic turnover reaction, for example lactate oxidase with an osmium-based redox hydrogel biosensor [24]. The principle may in principle also be attractive for non-enzymatic turnover reactions.

## Supplementary Information

Below is the link to the electronic supplementary material.

Derivation of the theoretical model, calculations, simulation equations and supplementary experiments are available in the Supplementary Information file.

Supplementary Material 1 (DOCX 2.71 MB)

## Data Availability

Original data will be made available upon reasonable request.
