# Supplementary material for: Zero-current chronopotentiometry for wired biosensors
Source: Mikrochim Acta. 2025 Dec 16;193(1):25. doi: 10.1007/s00604-025-07769-8 (PMC12708822; doi:10.1007/s00604-025-07769-8)
Supplement: Supplementary file 1 — Supplementary Material 1 (DOCX 2.71 MB) [file 604_2025_7769_MOESM1_ESM.docx]

**Supporting Information for:**

**Zero Current Chronopotentiometry for Wired Biosensors**

Andrea Nonis ^†*^, Polyxeni Damala ^†^, Eric Bakker ^†^*

University of Geneva, Department of Mineral and Analytical Chemistry, Quai-Ernest 30, 1205 Geneva (Switzerland)

Corresponding author: [Eric.Bakker@unige.ch](mailto:Eric.Bakker@unige.ch), [Andrea.Nonis@unige.ch](mailto:Andrea.Nonis@unige.ch)

Contributing author: [polina.damala@gmail.com](mailto:polina.damala@gmail.com)

# **Theorical Model**

Two reactions were considered, A) the enzymatic reaction of GOx and B) the electrons transfer reaction regenerating the GOx:

A) $Glucose+GOxFAD \underset{\to}{k_{ENZ}} Gluconolactone+GOxFADH_{2}$

B) $GOxFADH_{2}+2 {Fc}^{+} \underset{\to}{k_{ET}} GOxFAD+2 Fc+2 H^{+}$

Where *k_ENZ_* and *k_ET_* are the enzyme reaction rate and the electron transfer rate respectively. The oxidation of glucose generates 2 electrons, so 1 mol of glucose generates 2 mols of reduced ferrocene $Fc$.

***Membrane diffusion limiting case***

It was assumed that the diffusion limiting membrane, of thickness ($\delta_{m}$), allows to limit glucose flux ($J_{G}$) so that the rate limiting step is the mass transport and not the enzymatic reaction nor the electron transfer. This implies:

$$J_{G}\ll k_{ENZ}$$

$$k_{ET}\ll k_{ENZ}$$

With these assumptions, as soon as a molecule of glucose reaches the enzyme it will be converted to gluconolactone, and 2 ions of ferrocenium ${Fc}^{+}$ are converted to $Fc$. The resulting total reaction will be controlled by the slowest process.

$nFc=2nGlu$ (1)

Since the redox polymer will be first totally oxidized at *t* = 0, the total number of moles of ferrocene $n{Fc}^{T}$ is equal to the number of moles of oxidized ferrocenes ${nFc}^{+}$:

At *t* = 0 $n{Fc}^{T}={nFc}^{+}$ (2)

At *t* > 0, the total number of ferrocene moles is expressed as:

At *t* > 0 ${nFc}^{T}={nFc}^{+}+nFc$ (3)

due to conversion of ferrocenium ions by the enzymatic activity. Considering equation 1 and 2, the moles of oxidized ferrocene can be expressed by:

${nFc}^{+}={nFc}^{T}-2nGlu$ (4)

Since the electrode is covered by the redox polymer, it will dictate the potential at the electrode surface according to:

$E= E_{{Fc}^{+}/Fc}^{0}-\frac{RT}{F}ln\left( \frac{nFc}{{nFc}^{+}} \right)$ (5)

where $R$ is the perfect gas constant, $T$the temperature, $F$ the Faraday constant and $E_{{Fc}^{+}/Fc}^{0}$ the ferrocene couple redox potential. Using Fick’s 1^st^ law, the glucose flux can be calculated and therefore the number of moles of glucose reaching the enzyme redox layer with time:

$J_{G}=K_{m}\frac{c_{Glu}}{\delta_{m}}$ (6)

$nGlu= J_{G}At$ (7)

$nGlu=K_{m}\frac{c_{Glu}}{\delta_{m}}At$ (8)

where $c_{Glu}$ is the bulk glucose concentration, *A* the electrode area, $t$ the time and $K_{m}$ the membrane constants. The membrane constant is equal to $K_{m}=P_{m}D_{m}$ where $P_{m}$ is the glucose partition coefficient between the solution and membrane phase and $D_{m}$ the glucose diffusion coefficient in the membrane. $nFc$ and ${nFc}^{+}$ can be expressed as a function of time:

$nFc=2nGlu=2K_{m}\frac{c_{Glu}}{\delta_{m}}At$ (9)

$n{Fc}^{+}= {nFc}^{T}-2K_{m}\frac{c_{Glu}}{\delta_{m}}At$ (10)

In the experiments, the transition time $\tau$ correspond to when all the ${Fc}^{+}$ is converted into $Fc$*.* When $t=\tau$, ${nFc}^{+}=0$, so:

$0={nFc}^{T}-2K_{m}\frac{c_{Glu}}{\delta_{m}}A\tau$ (11)

$c_{Glu}$ can be related to $\tau$ by:

$\frac{1}{\tau}=\frac{2K_{m}A}{n{Fc}^{T}\delta_{m}}c_{Glu}$ (12)

The potential response of the sensor with time can be written by incorporating equations (9) and (10) into (5):

$E= E_{{Fc}^{+}/Fc}^{0}-\frac{RT}{F}\left( \frac{2K_{M}\frac{c_{Glu}}{\delta_{m}}At}{{nFc}^{T}-2K_{m}\frac{c_{Glu}}{\delta_{m}}At} \right)$ (13)

***No diffusion limiting membrane case***

The case without diffusion limiting membrane can be treated in a similar manner. It is important to assume that the enzyme is still not rate limiting so that we are still in the linear range of Michaelis-Menten plot. The glucose concentration will deplete at the electrode surface with time due to enzymatic activity and result in an increasing glucose gradient in the aqueous phase at the sensor surface. This creates an aqueous diffusion distance that increases with time in agreement with Cottrellian behavior:

$\delta_{aq}=2\sqrt{D_{aq}t}$ (14)

where $D_{aq}$ is the diffusion coefficient of glucose in water. The same relationships used above are considered:

$n{Fc}^{T}=n{Fc}^{+}+nFc$ (15)

$nFc=2nGlu$ (16)

$J_{G}=D_{aq}\frac{c_{Glu}}{\delta_{aq}}=D_{aq}\frac{c_{Glu}}{2\sqrt{D_{aq}\Delta t}}=\frac{\sqrt{D_{aq}}c_{Glu}}{2\sqrt{t}}$ (18)

An expression of the number of moles as a function of time is obtained:

$nFc=2J_{G}At=\frac{2\sqrt{D_{aq}}c_{Glu}}{2\sqrt{t}}At=c_{Glu}A\sqrt{D_{aq}t}$ (19)

${nFc}^{+}={nFc}^{T}-\frac{2\sqrt{D_{aq}}c_{Glu}}{2\sqrt{t}}At={nFc}^{T}-c_{Glu}A\sqrt{D_{aq}t}$ (20)

Similarly, to the diffusion limiting case, when $t=\tau$, ${nFc}^{+}=0$ :

$0={nFc}^{T}-c_{Glu}A\sqrt{D_{aq}t}$ (21)

$c_{Glu}$ can be related to $\tau$ by:

$\frac{1}{\sqrt{\tau}}=\frac{A\sqrt{D_{aq}}}{{nFc}^{T}}c_{Glu}$ (22)

The potential response of the sensor over time is found by inserting equation (18) and (19) into (5):

$E= E_{{Fc}^{+}/Fc}^{0}-\frac{RT}{F}ln\left( \frac{c_{Glu}A\sqrt{D_{aq}t}}{{nFc}^{T}-c_{Glu}A\sqrt{D_{aq}t}} \right)$ (23)

# **Results:**

***Flow cell***


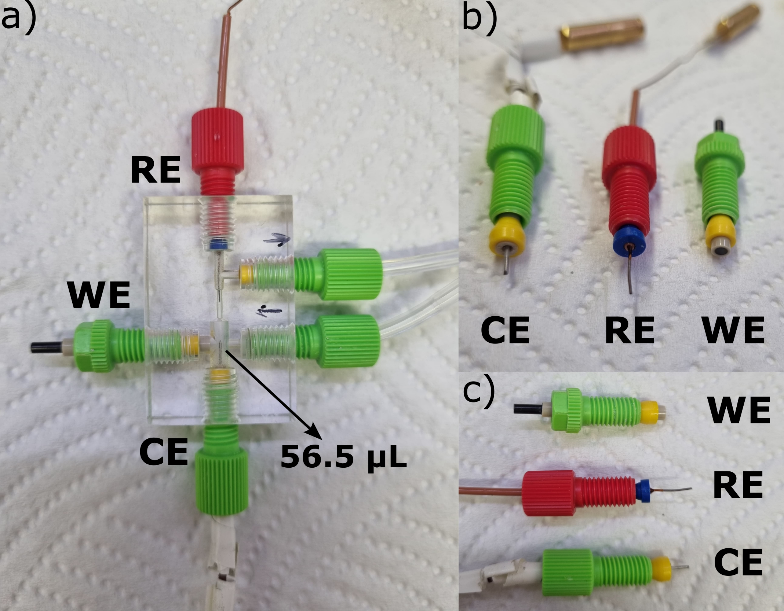


**Fig. S1**: a) Flow cell set up. b) Working (WE), counter (CE) and reference (RE) electrode front view. c) Working (WE), counter (CE) and reference (RE) electrodes side view. (WE: in-house made glassy carbon electrode, CE: Pt wire, RE: Ag/AgCl wire).

***Optimization of the biosensor***


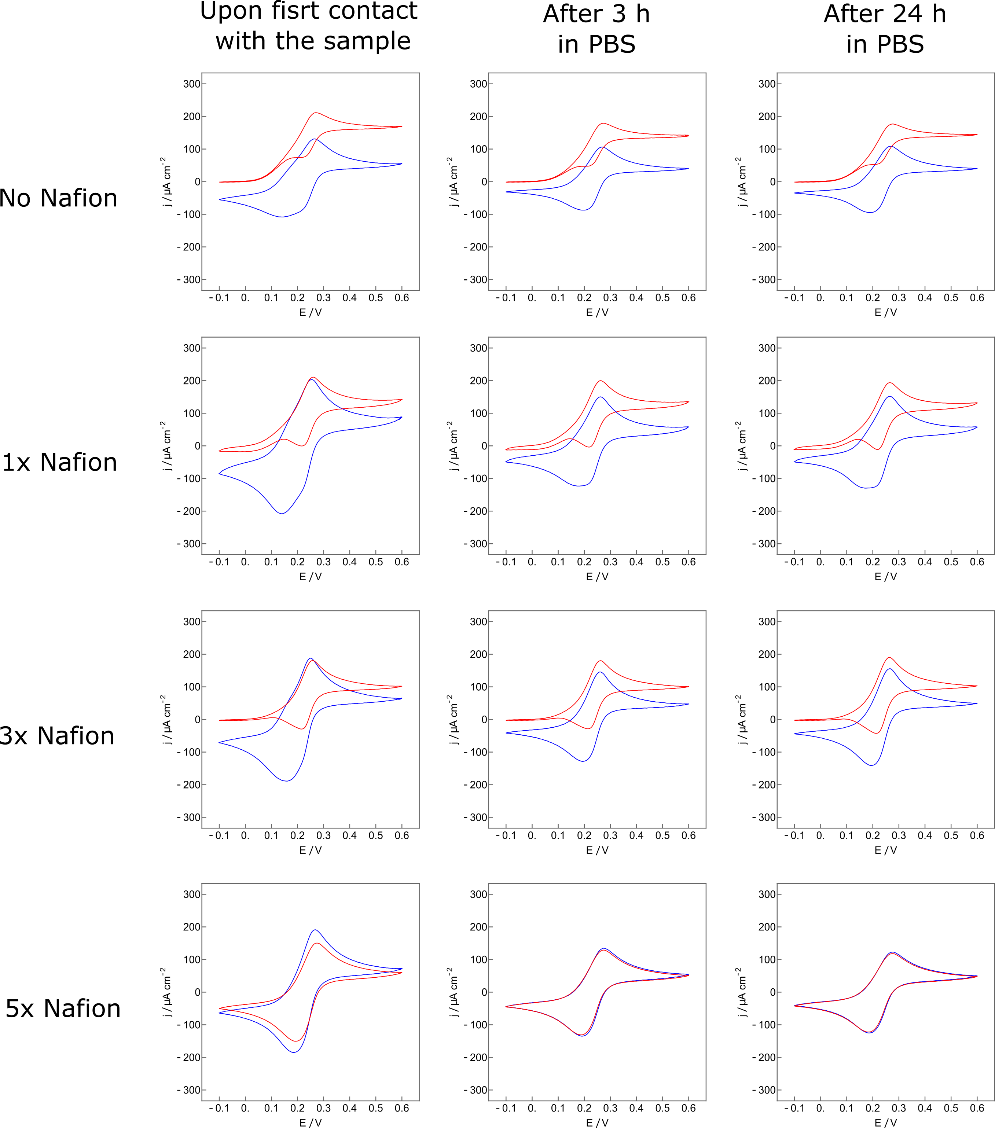


**Fig. S2**: Cyclic voltammograms of the biosensor (Fc-C_6_-bPEI/GOx/cross-linker) with no Nafion layer, 1, 3 and 5 Nafion layer deposited on the biosensor (top to bottom row) upon contact with PBS, after 3 hours in PBS and 24 hours in PBS (left to right column) in PBS (blue) and in 10 mM glucose. The last scan of 10 scans is showed here. (ΔE= -0.100 to 0.600 V, 0.050 V s^-1^)

An increase of current is observed with 10 mM glucose due to the enzymatic activity in all measurements, except with five Nafion layers coatings (Figure S2, bottom row). This indicates that glucose does not permeate through the membrane sufficiently. With three layers, glucose diffuses across the membrane and sufficient diffusion limiting properties are maintained. A three Nafion layers coating was used as diffusion limiting membrane in subsequent experiments.


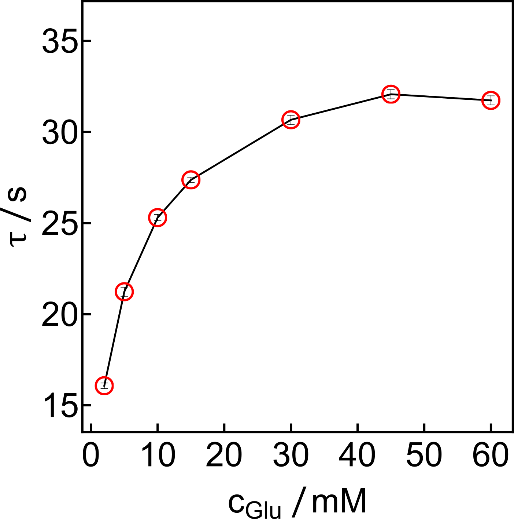


**Fig. S3**: Transition time recorded for different oxidation times with a three-layer Nafion membrane biosensor (Fc-C_6_-bPEI/GOx/crosslinker + 3 layers Nafion) at an applied potential of 0.400 V in 4 mM glucose.

From the data in Figure S3 an oxidation time of 45 s was maintained for all chronopotentiometric measurements.

***Characterization of the biosensor***


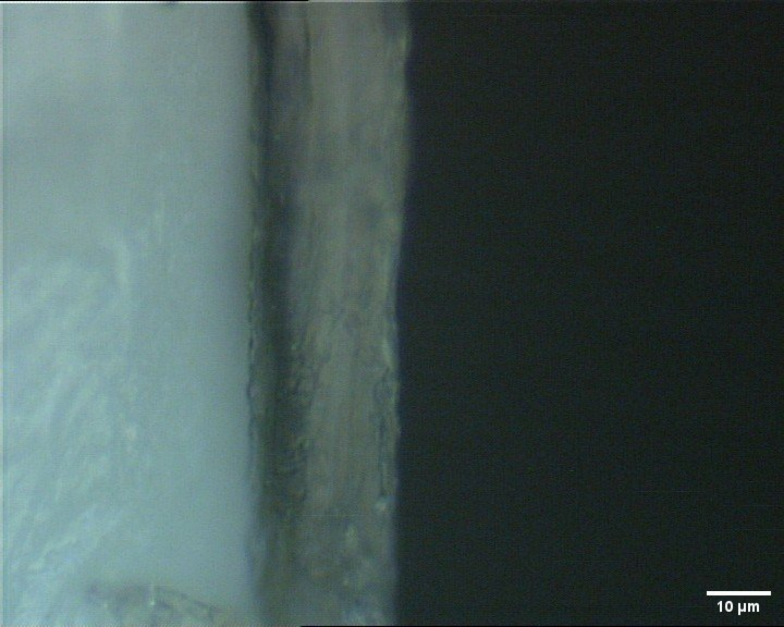


***Fig. S4****: 50x magnified Nafion membrane cross section picture.*

The Nafion membrane thickness was measured at 23.0 ± 0.9 µm.

Using previously reported by our group 2 µL of Fc-C_6_-bPEI/GOx/cross-linker thickness (22.5 ± 3.0 µm), the electrode area (0.0314 cm^2^), and the theorical ferrocene concentration (1.49 M), the theorical number of ferrocene moles was calculated as 101 nmol (corresponding to a 9.7 mC charge) [1].


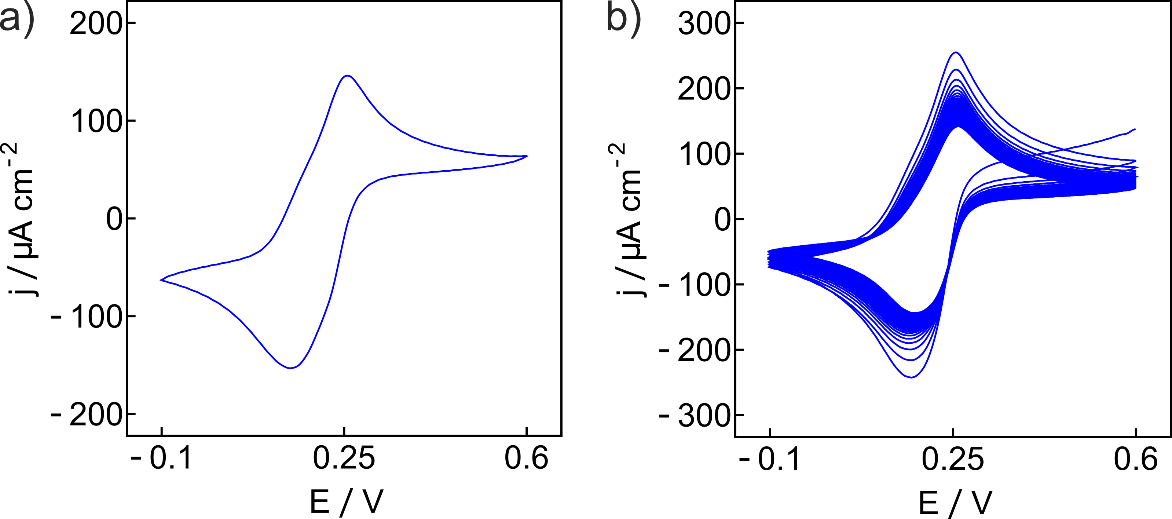


**Fig. S5**: a) Cyclic voltammogram of the biosensor (Fc-C_6_-bPEI/GOx/PEGDE + 3 layers Nafion) in constant flow PBS upon first contact with the solution. The last scan over 15 scans is presented (255 µL min^-1^, 0.050 V s^-1^). b) 50 cyclic voltammograms scans stability of the biosensor in constant PBS flow. (510 µL min^-1^, ΔE = -0.100 V to 0.600 V, 0.050 V s^-1^)

The redox potential of the Fc-C_6_-bPEI/GOx/cross-linker was measured in PBS by cyclic voltammetry (0.050 V s^-1^) as 0.204 ± 0.001 V (vs Ag/AgCl), observing a peak-to-peak separation of 0.102 ± 0.001 V. The electrochemically addressable amount of ferrocene, 0.11 and 0.15 nmol, were obtained by integrating the area under the anodic peak and cathodic peak respectively. This represents an average of just 0.13 % of the theorical ferrocene moles calculated from the degree of substitution. With the sensing layer volume, a concentration of 1.97 mM (1.97 x 10^-6^ mol cm^-3^) of active redox centers is then obtained. This concentration was considered the following calculations.


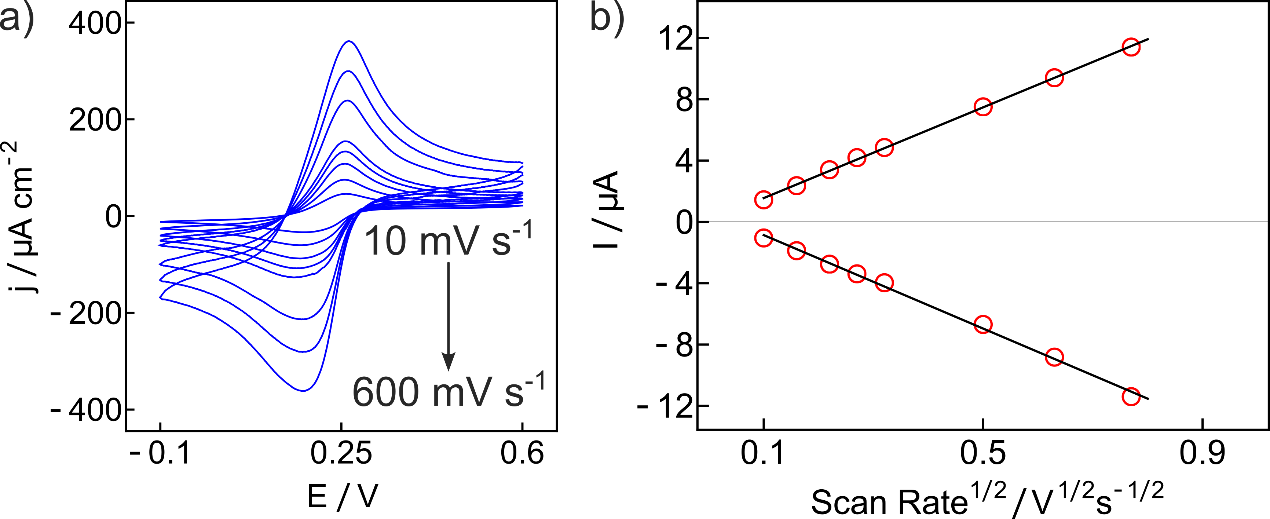


**Fig. S6**: Scan rate study under constant flow (255 µL min^-1^) of the biosensor (Fc-C_6_-bPEI/GOx/PEGDE + 3 layers Nafion) in PBS (ΔE = -0.100 V to 0.600 V). a) Last scan of 2 scans. b) Randels-Sevcik plot.

A Randels-Sevcik plot yield an anodic peak slope of 1.468 ± 0.015 x 10^-5^ A s^1/2^ V^-1/2^ and -1.512 ± 0.031 x 10^-5^ A s^1/2^ V^-1/2^ cathodic peak slope were obtained and used to calculate the apparent electrons diffusion coefficient with:

$i_{p}=2.69 x 10^{5} n^{3/2}AD_{e}^{1/2}c_{R}v^{1/2} \to D_{e}=\left( \frac{s}{2.69x{10}^{5} Ac_{R}n^{3/2}} \right)^{2}$ (24)

Where *s* is the slope, *A* the electrode area, *c_R_* the concentration of redox centers and *n* the number of electrons involved. With *A* = 0.031 cm^2^, *c_R_* = 1.97 x 10^-6^ mol cm^-3^ and *n* = 1, an average *D_e_* of 8.75 ± 0.27 x 10^-7^ cm^2^ s^-1^ was obtained. The error was calculated using the error of both slopes ($\Delta S$) to find the error $\Delta D_{e}$:

$D_{e}=\left( \frac{s}{2.69x{10}^{5} Ac_{R}n^{3/2}} \right)^{2}\to D_{e}=s^{2}\left( \frac{1}{2.69x{10}^{5} Ac_{R}n^{3/2}} \right)^{2}\to{\Delta D}_{e}=2s\left( \frac{1}{2.69x{10}^{5} Ac_{R}n^{3/2}} \right)^{2}\Delta s$ (25)

This value was confirmed by recording the charging current of the biosensor (Fc-C_6_-bPEI/GOx/PEGDE + 3 Nafion layers) in PBS. (0.400 V applied for 45 seconds) (Fig. S7).


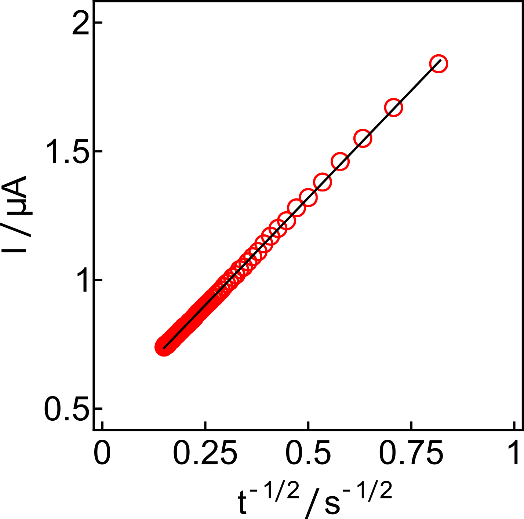


**Fig. S7**: Cottrell plot (i = f(t^-1/2^)) of the biosensor (Fc-C_6_-bPEI/GOx/PEGDE + 3 Nafion layers) in PBS. (E = 0.400 V, t = 45 s)

A similar result, 2.68 ± 0.47 x 10^-7^ cm^2^ s^-1^, was obtained from the slope (1.667 ±0.003 x 10^-6^ A s^1/2^) fitting the current data with Cottrell equation ($i=f(t^{-\frac{1}{2}})$) with the same parameters as the scan rate study according to:

$i=\frac{nFAc_{R}D_{e}^{1/2}}{\pi^{1/2}t^{1/2}} \to D_{e}=\left( \frac{s\pi^{1/2}}{nFAc_{R}} \right)^{2}$ (26)

Where $F$ is the Faraday’s constant and $t$ the time. The error was calculated using the error on the slope. These values are in the same order of magnitude as previously reported values for ferrocene based redox polymers [2–5].

***Table S1****: Average oxidation charges of the glucose “zero-current” chronopotentiometric calibration.*

| c_Glu_ / mM | Oxidation Charge / µC | Relative error / % |
| --- | --- | --- |
| 3 | 22.24 | 0.64 |
| 4 | 25.00 | 0.18 |
| 5 | 27.25 | 0.22 |
| 6 | 30.62 | 0.10 |
| 7 | 32.78 | 0.16 |
| 8 | 34.83 | 0.11 |
| 9 | 36.76 | 0.22 |
| 10 | 38.21 | 0.18 |
| 12 | 41.13 | 0.06 |
| 15 | 45.20 | 0.03 |
| 20 | 50.72 | 0.03 |


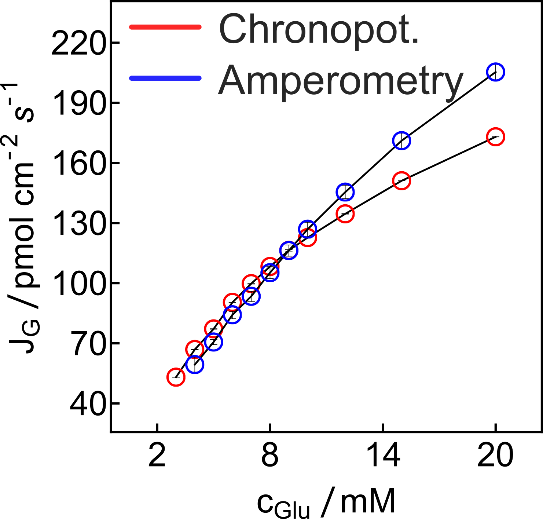


***Fig. S8:*** *Glucose fluxes calculated from the oxidation current from the zero-current chronopotentiometry calibration (red) and from the amperometry calibration (blue).*

***Table S2:*** *Glucose flux calculated from the oxidation step of the zero-current chronopotentiometric and amperometric glucose calibration.*

|  | Zero-current chronopotentiometric calibration | | Amperometric calibration | |
| --- | --- | --- | --- | --- |
| c_Glu_ / mM | **Glucose flux / pmol cm^-2^ s^-1^** | **Relative error / %** | **Glucose flux / pmol cm^-2^ s^-1^** | **Relative error / %** |
| 3 | 53.19 | 0.05 |  |  |
| 4 | 66.92 | 0.18 | 59.28 | 6.28 |
| 5 | 77.17 | 0.16 | 70.63 | 1.78 |
| 6 | 90.43 | 0.08 | 84.22 | 2.08 |
| 7 | 99.77 | 0.20 | 93.46 | 2.70 |
| 8 | 108.33 | 0.14 | 105.23 | 2.75 |
| 9 | 116.37 | 0.33 | 116.29 | 2.41 |
| 10 | 122.76 | 0.23 | 126.93 | 2.73 |
| 12 | 134.61 | 0.10 | 145.43 | 2.22 |
| 15 | 151.12 | 0.03 | 171.11 | 2.18 |
| 20 | 173.05 | 0.03 | 205.37 | 2.09 |

The fluxes were obtained from the steady state current observed during the oxidation steps in “zero-current” chronopotentiometry and during the amperometric calibration for each concentration. The flux of glucose was calculated using:

$J_{G}= \frac{I_{steady}}{nFA}$ (27)

Where n = 2 and $I_{steady}$ the measured steady state current. The obtained charges are above the ferrocene charge since glucose is present during the oxidation and so contributing to the current.


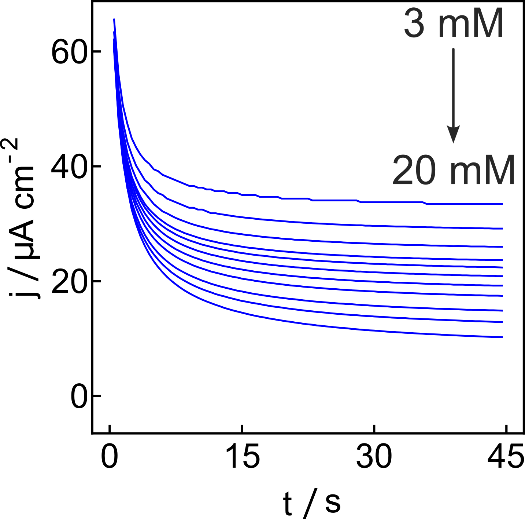


***Fig. S9****: Oxidation step currents of the “zero-current” chronopotentiometric measurements form 3-20 mM glucose. (E= 0.400 V, t = 45 s).*


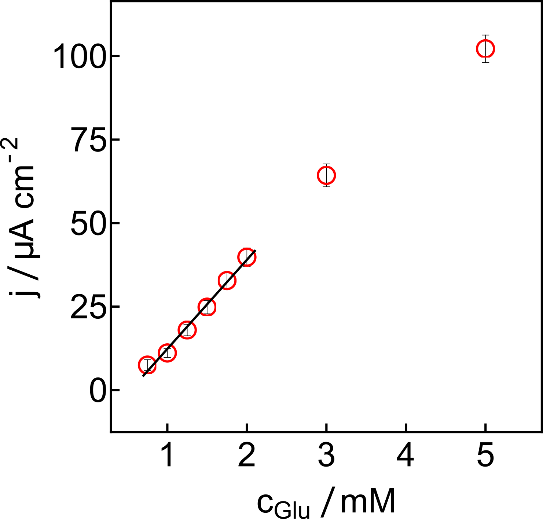


***Fig. S10****: Chronoamperometric calibration of the biosensor without the Nafion membrane. (E = 0.400 V)*

***Table S3:*** *Transition times observed from the “zero-current” chronopotentiometry glucose calibration, from the constant current chronopotentiometry applying the calculated equivalent currents from flux and the calculated transition times form equation 12.*

| c_Glu_ / mM | τ / s | | |
| --- | --- | --- | --- |
|  | **Zero-current chronopotentiometry** | **Relative error / %** | **Calculated / s** |
| 3 | 71.1 | 1.99 | 28.7 |
| 4 | 36.7 | 0.55 | 21.5 |
| 5 | 26.5 | 0.78 | 17.2 |
| 6 | 19.4 | 0.60 | 14.3 |
| 7 | 15.7 | 0.64 | 12.3 |
| 8 | 13.3 | 0.44 | 10.8 |
| 9 | 11.6 | 0.50 | 9.6 |
| 10 | 10.2 | 0.56 | 8.6 |
| 12 | 8.5 | 0.68 | 7.2 |
| 15 | 6.9 | 0.84 | 5.7 |
| 20 | 5.4 | 1.08 | 4.3 |

***Simulation***

The system was simulated using measured experimental parameters and constants:

A = 0.03141 cm^2^

$c_{Fc+}$ = 1.97 mM

z = 1

F = 96485 C mol^-1^

$K_{m}$ = 1.12 x 10^-7^ cm^2^ s^-1^

$K_{pot}$ = 10^-10^

$$x_{max}= \frac{\delta_{P}}{\delta_{step}}$$

$\delta_{P}$ = 22.5 µm

$\delta_{step}$ = 2.25 µm

$$t_{max}=\frac{\tau}{\delta_{t}}$$

$\delta_{t}$ = 0.01 τ

$$Tau=\frac{K_{m}\delta_{t}}{\delta_{step}^{2}}$$

***Table S4:*** *Oxidation current recorded during the oxidation step of the zero-current chronopotentiometry used for the simulation.*

| c_Glu_ / mM | I_Ox_ / µA |
| --- | --- |
| 3 | 0.322 |
| 4 | 0.406 |
| 5 | 0.468 |
| 6 | 0.548 |
| 7 | 0.605 |
| 8 | 0.657 |
| 9 | 0.706 |
| 10 | 0.744 |
| 12 | 0.816 |
| 15 | 0.916 |
| 20 | 1.049 |

The system was modeled using the finite element analysis for a one-dimensional diffusional problem [6,7]. Concentration change in the bulk film treated as Fick’s diffusion with a concentration change through the elements and time being:

$c_{Fc+}\left( x, t \right)=c_{Fc+}\left( x, t-1 \right)+Tau\left[ c_{Fc+}\left( x-1, t-1 \right)-2c_{Fc+}\left( x, t-1 \right)+c_{Fc+}\left( x+1, t-1 \right) \right]$ (28)

The boundary conditions were set as for electrode surface element as an inert boundary (Eq. 29) (considered to be position 0, described by the reflection of concentration) and the polymer/Nafion interface (considered as position $x_{max}$) by solving relationship of the applied current and the species flux (Eq.31):

$c_{Fc+}\left( 0, t \right)=c_{Fc+}\left( 0, t-1 \right)+2Tau\left[ c_{Fc+}\left( 1, t-1 \right)-c_{Fc+}\left( 0, t-1 \right) \right]$ (29)

$i= -zFA\frac{K_{m}}{\delta_{step}}\frac{c_{Fc+}\left( x_{max} \right)\left[ c_{Fc+}\left( x_{max} \right)-c_{Fc+}\left( x_{max}-1 \right) \right]}{c_{Fc+}\left( x_{max} \right)+K_{pot}}$ (30)

$\longrightarrow-\frac{i\left[ c_{Fc+}\left( x_{max} \right)+K_{pot} \right]}{zAK_{m}c_{Fc+}\left( x_{max} \right)}=c_{Fc+}\left( x_{max} \right)-c_{Fc+}\left( x_{max}-1 \right)$ (31)

with: $K_{pot}=K_{sel}^{\frac{O}{P}}{(\frac{c_{O}}{c_{P}})}^{zo/zP}$ (32)

The simulation is let run in the time interval corresponding to the transition time experimental observed. Each concentration line corresponds to a time interval of 0.1 τ.


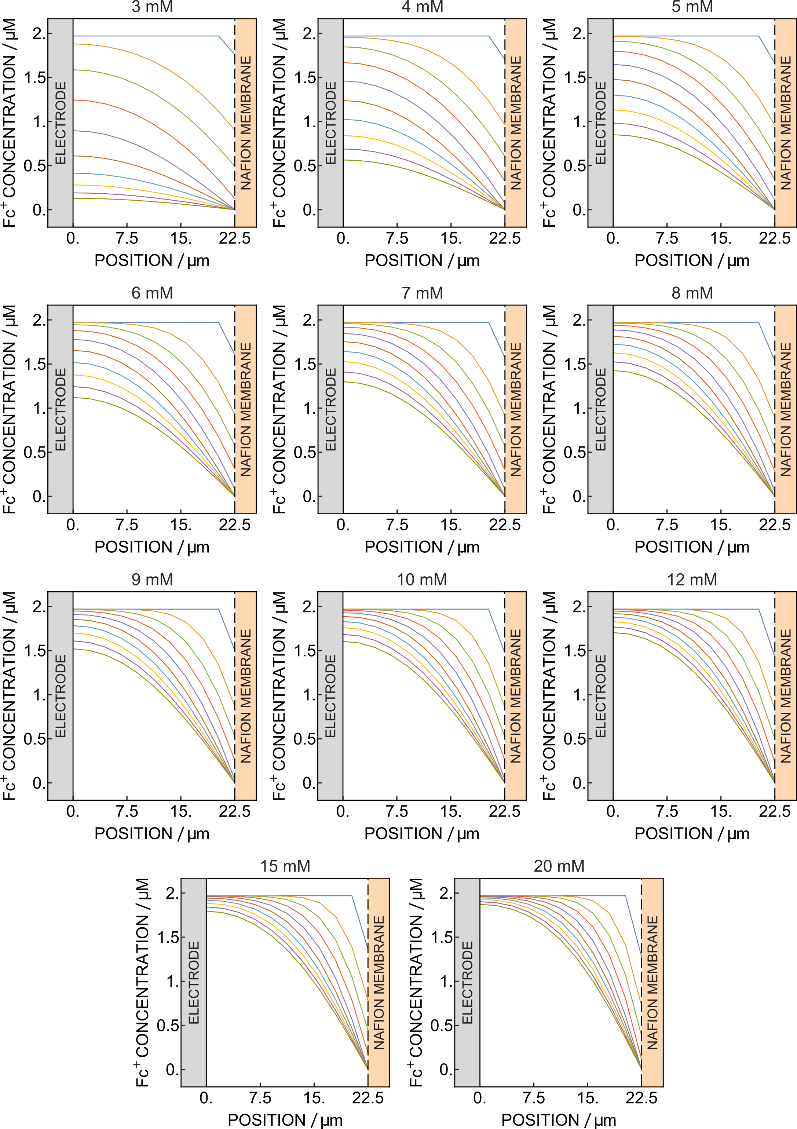


***Fig. S11****: Simulated zero-current chronopotentiometric Fc^+^ concentration profile inside the sensing layer.*


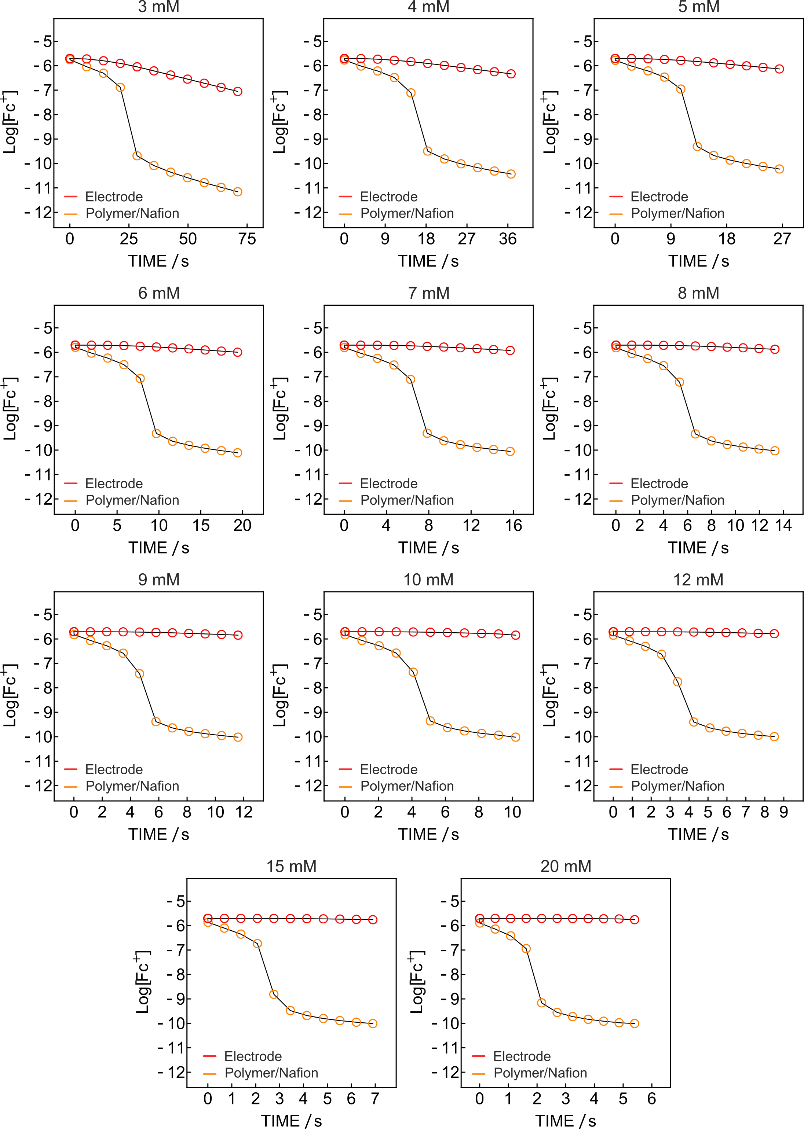


***Fig. S12:*** *Logarithm of the Fc^+^ simulated concentration at the polymer/Nafion interface (orange) and at the electrode surface (red).*

# References:

1. Damala P, Tiuftiakov NYu, Bakker E (2024) Avoiding Potential Pitfalls in Designing Wired Glucose Biosensors. ACS Sens 9 (1): 2–8. <https://doi.org/10.1021/acssensors.3c01960>

2. Chuang CL, Wang YJ, Lan HL (1997) Amperometric glucose sensors based on ferrocene-containing B-polyethylenimine and immobilized glucose oxidase. Anal Chim Acta 353 (1): 37–44. <https://doi.org/10.1016/S0003-2670(97)00372-3>

3. Mao F, Mano N, Heller A (2003) Long Tethers Binding Redox Centers to Polymer Backbones Enhance Electron Transport in Enzyme “Wiring” Hydrogels. J Am Chem Soc 125 (16): 4951–4957. <https://doi.org/10.1021/ja029510e>

4. Milton RD, Hickey DP, Abdellaoui S, Lim K, Wu F, Tan B, Minteer SD (2015) Rational design of quinones for high power density biofuel cells. Chem Sci 6 (8): 4867–4875. <https://doi.org/10.1039/C5SC01538C>

5. Bu HZ, English AM, Mikkelsen SR (1997) Charge Transport in Ferrocene-Containing Polyacrylamide-Based Redox Gels. J Phys Chem B 101 (46): 9593–9599. <https://doi.org/10.1021/jp9722263>

6. Bakker E (2014) Fundamentals of Electroanalysis, Geneva.

7. Bard AJ, Faulkner LR (2001) Electrochemical methods: fundamentals and applications. 2nd edn. Wiley, New York Weinheim.
